# Proposal of a Nomogram for Predicting Survival in Patients with Siewert Type II Adenocarcinoma of the Esophagogastric Junction After Preoperative Radiation

**DOI:** 10.1245/s10434-019-07237-7

**Published:** 2019-02-25

**Authors:** Feng Liu, Rui Zhou, Funeng Jiang, Guolong Liu, Kangbao Li, Guodong Zhu

**Affiliations:** 1 Department of Geriatrics, National Clinical Key Specialty, Guangzhou First People’s Hospital, School of Medicine, South China University of Technology, Guangzhou, 510180 Guangdong China; 2 Department of Oncology, Nanfang Hospital, Southern Medical University, Guangzhou, 510515 China

## Abstract

**Background:**

Preoperative radiotherapy tends to be more frequently used for patients with adenocarcinoma of the esophagogastric junction (AEG); however, the prognostic values of postoperative pathologic characteristics in these patients remain unclear. This study aimed to examine the outcomes in Siewert type II AEG patients receiving preoperative radiotherapy to identify the predictive factors for overall survival (OS).

**Methods and Results:**

A total of 1818 AEG patients undergoing preoperative radiotherapy were reviewed. Univariate analyses showed that age, sex, histology, tumor grade, positive lymph node (PLN), lymph node ratio, and log odds of positive lymph nodes (LODDS) were significantly correlated with OS; however, only age, grade, PLN, and LODDS were identified as independent risk factors in a multivariate regression model. Subsequently, patients were randomly grouped into training and validation cohorts (1:1 ratio), and the beta coefficients of these variables in the training set were used to generate the nomogram. The composite nomogram showed improved prognostic accuracy in the training, validation, and entire cohorts compared with that of TNM stage alone.

**Conclusions:**

In conclusion, our proposed nomogram represents a promising tool for estimating OS in Siewert type II AEG patients after preoperative radiotherapy.

**Electronic supplementary material:**

The online version of this article (10.1245/s10434-019-07237-7) contains supplementary material, which is available to authorized users.

The incidence of adenocarcinoma of the esophagogastric junction (AEG) has increased dramatically in both Western and Asian countries over the past several decades.^1^^–^3 Complete surgical removal represents the only curative treatment for patients with AEG.^4^^,^^5^ The surgical requirements for an eligible resection include surgery with curative intent and en bloc resection of the tumor with both macroscopically and microscopically negative margins (R0 resection). However, at diagnosis, most Siewert type II AEG tumors have infiltrated deep into the gastric wall and metastasized to the lymph nodes (LNs) and distant organs, i.e. advanced carcinoma.^6^^–^8 Under such circumstances, various treatment regimens attempting to improve patient survival, including preoperative radiotherapy, have been developed.

The survival impact of Siewert type II AEG in goblet cell carcinoma (GCC) has been explored in several studies.^9^^–^13 Although few studies have been statistically powered and the survival benefit remains controversial, preoperative radiotherapy combined with chemotherapy rather than preoperative chemotherapy alone is more effective for achieving good pathologic response.^14^ Indeed, preoperative radiotherapy can achieve clinical downstaging before resection and can reduce the burden of residual microscopic disease at surgery, thus increasing the possibility of R0 resection.^15^^,^^16^ As a result, the combination of preoperative radiotherapy and chemotherapy tends to be more frequently used, rather than chemotherapy alone, for advanced Siewert type II AEG patients in the US.^17^ However, the prognostic values of various postoperative pathologic characteristics in Siewert type II AEG patients undergoing preoperative radiotherapy remain unclear. Hence, the objective of this retrospective study from the Surveillance, Epidemiology, and End Results (SEER) database was to identify the factors that affect overall survival (OS) in Siewert type II AEG patients receiving preoperative radiotherapy. Moreover, we also aimed to identify the best staging systems for determining the prognosis of these patients.

## Materials and Methods

### Patients

Data were obtained from the SEER public database. In the 18 SEER registries, the 2016 release of the public use dataset from 1998 and 2013 was queried to identify Siewert type II AEG patients with microscopic diagnostic confirmation. Although the SEER database did not provide detailed information on the Siewert type classification for AEG, the combined selection terminology of ‘Primary Site’ encoded 160 (Cardia) and ‘CS site-specific factor 25’ encoded 982 (esophagus, gastroesophageal junction), allowed us to obtain the Siewert type II AEG.^18^ Patients were included if they were M0 stage and had received preoperative radiotherapy, surgery, and pathologic examination of the LNs. The National Cancer Institute’s SEER*Stat software (Surveillance Research Program, National Cancer Institute SEER*Stat software [www.seer.cancer.gov/seerstat], Version 8.1.5) was used to access the database.

### Demographic and Clinicopathological Variables

Patient demographic and clinicopathological variables were retrieved from the SEER database. The LN metastasis variables we analyzed in the present study included the positive LN (PLN) count, LN ratio (LNR) value, and log odds of positive LNs (LODDS) value. The LNR value was defined as the PLN count divided by the total number of examined LNs (ELNs),^19^^,^^20^ while the LODDS value was defined as log_e_ ([PLN + 0.5]/[negative LN + 0.5]).^21^^,^^22^ The classification intervals of the PLN count were consistent with the N classification in the 8th edition of the American Joint Committee on Cancer (AJCC) staging system,^23^^,^^24^ stratified as ypN0 (no PLNs), ypN1 (one to two PLNs), ypN2 (three to six PLNs), and ypN3 (more than seven PLNs). The final subgroup definitions of the demographic and clinicopathological variables are shown in electronic supplementary Table S1.

Nomograms were established based on multivariate Cox regression coefficients, and calibration plots were generated to compare the expected and observed survival probabilities. Decision curve analyses (DCAs) were performed to assess the clinical utility of prediction models by quantifying the net benefits when different threshold probabilities were considered. In general, the strategy with the highest net benefit at any given risk threshold was considered to have the highest clinical value.

### Statistical Analysis

All statistical analyses were conducted using R software (R Foundation for Statistical Computing, Vienna, Austria) and SPSS software version 19.0 (IBM Corporation, Armonk, NY, USA). Missing values were handled by complete case analyses. Group comparisons were performed for continuous and categorical variables using the one-way analysis of variance (ANOVA) and Chi square test, respectively. The secondary endpoint was OS, defined as the period from diagnosis to death due to any reason. The univariate and multivariate analyses were performed using the Cox regression model 30 with ‘Forward LR’ methods to investigate which clinicopathological factors significantly correlated with OS. Among these factors, age, sex, ethnicity, ypT category, tumor grade, and tumor histology type were entered into the regression models as categorical variables; the PLN, LNR, and LODDS were analyzed in the form of continuous variables. Hazard ratios (HRs) and 95% confidence intervals (CIs) were calculated, with an HR < 1.0 indicating a survival benefit. The Cox regression coefficients were employed to generate nomograms, and discrimination of the prognostic models was measured and compared using Harrell’s concordance index (C-index) with the ‘survival’ package. Calibration plots were generated to compare the expected and observed survival probabilities. DCAs were performed to assess the clinical utility of prediction models by quantifying the net benefits when different threshold probabilities were considered. In general, the strategy with the highest net benefit at any given risk threshold was considered to have the highest clinical value. Akaike Information Criterion (AIC) analyses^25^^,^^26^ were used to compare the performances among different LN classifications and staging systems. A smaller AIC value indicated a more desirable model for predicting the outcome. All statistical tests performed were two-sided and *p* values < 0.05 were considered statistically significant. This study was conducted and reported in line with the Transparent Reporting of a multivariate prediction model for Individual Prediction or Diagnosis (TRIPOD) guidelines.

## Results

### Patient Selection and Characteristics

**Fig. 1:**
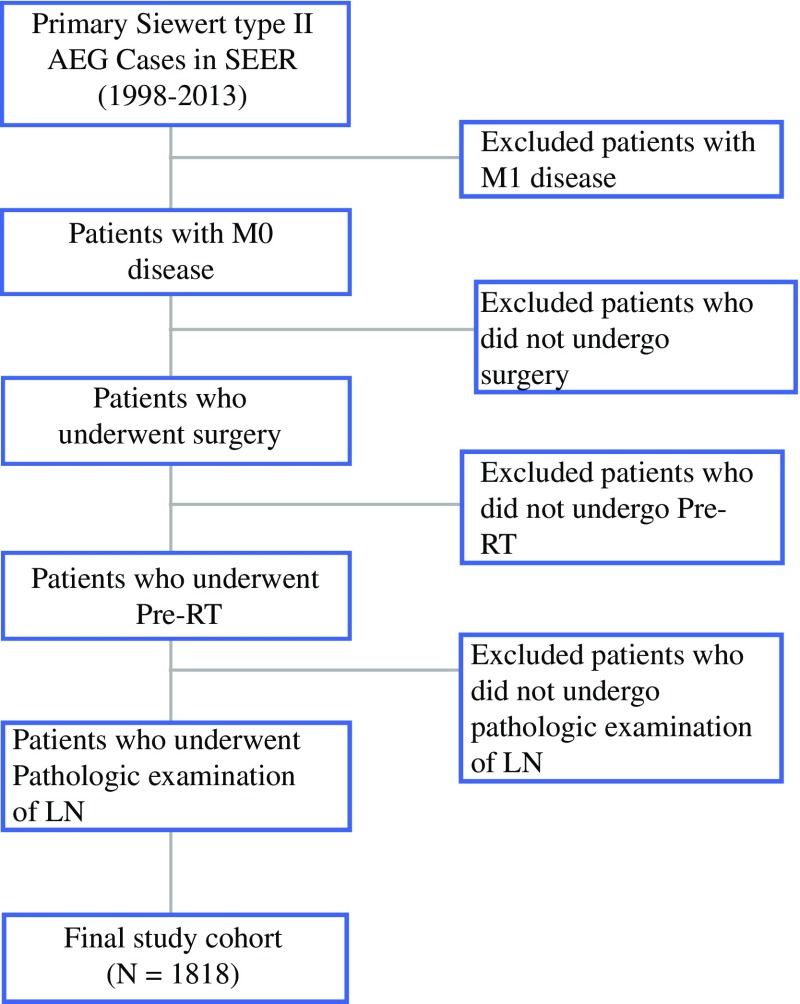
Selection of patients included in the study. *AEG* adenocarcinoma of the esophagogastric junction, *SEER* Surveillance, Epidemiology, and End Results database, *Pre*-*RT* preoperative radiotherapy, *LN* lymph node

The patient selection schema is shown in Fig. 1. Overall, a total of 1818 patients were included in this study (see electronic supplementary Table S2 for detailed patient characteristics). The median age at diagnosis was 62.0 years (range 24–86). Throughout the follow-up period, there were 997 deaths, 738 of which were attributable to GCC.

### Survival Analysis of Postoperative Prognostic Factors

In the univariate analyses of clinicopathological variables as prognostic factors for OS, age, sex, tumor grade, histology type, and all three LN-related factors (PLN, LNR, and LODDS classifications) were significant risk factors for Siewert type II AEG patients after preoperative radiotherapy, whereas the ypT category did not correlate with OS (Table 1). In multivariate analyses of all patients (Table 1), only age, tumor grade, PLN count, and LODDS value were significantly associated with OS.

**Table 1:** Univariate and multivariate survival analyses

Item	UVA of all patients		MVA of all patients		MVA of the training cohort	
	HR (95% CI)	*p* value	HR (95% CI)	*p* value	HR (95% CI)	*p* value
Age, years (vs. ≤ 65 years)	1.46 (1.28–1.68)	< 0.01	1.27 (1.11–1.46)	< 0.01	1.28 (1.07–1.52)	< 0.01
Sex (vs. male)	0.81 (0.67–0.99)	0.04	NA	NA		
Race (vs. White)	0.90 (0.68–1.19)	0.44	NA	NA		
ypT category (vs. ypT1)		0.21	NA	NA		
ypT2	0.91 (0.71–1.15)		NA	NA		
ypT3	1.04 (0.84–1.30)		NA	NA		
ypT4	1.10 (0.88–1.37)		NA	NA		
Histology (vs. unspecified AC)		0.02	NA	NA		
SRC	1.33 (1.10–1.62)	< 0.01	NA	NA		
ITAC	0.95 (0.70–1.29)	0.74	NA	NA		
MAC	0.94 (0.69–1.29)	0.72	NA	NA		
Other	1.26 (0.99–1.59)	0.06	NA	NA		
Grade (vs. grade 1/2)	1.46 (1.28–1.68)	< 0.01	1.31 (1.14–1.51)	< 0.01	1.32 (1.10–1.58)	< 0.01
PLN^a^	1.01 (1.00–1.01)	< 0.01	1.03 (1.01–1.06)	0.01	1.03 (0.99–1.06)	0.06
LNR^a^	3.54 (2.85–4.40)	< 0.01	NA	NA		
LODDS^a^	1.27 (1.22–1.33)	< 0.01	1.21 (1.14–1.28)	< 0.01	1.16 (1.08–1.25)	< 0.01

*HR* hazard ratio, *CI* confidence interval, *AC* adenocarcinoma, *PLN* positive lymph node, *LNR* lymph node ratio, *LODDS* log odds of positive lymph nodes, *NA* not available, *UVA* univariate analysis, *MVA* multivariate analysis, *SRC* signet ring carcinoma, *ITAC* intestinal-type adenocarcinoma, *MAC* mucinous adenocarcinoma

^a^Continuous variables

### Characteristics of Patients in the Training and Validation Cohorts

Patients were randomly split into derivation and validation sets based on a 6:4 ratio. Comparison of these two patient groups showed no significant baseline differences (all *p* > 0.05) (Table 2). The training group was used to evaluate the prognostic effect of clinicopathological variables to determine the beta coefficients (*β*) (Table 3). Age > 65 years compared with age ≤ 65 years (*β* = 0.24, *p* < 0.01), grade 3–4 compared with grade 1–2 (*β* = 0.27, *p* < 0.01), PLN number (*β* = 0.03, *p* = 0.06), and LODDS value (*β* = 0.15, *p* < 0.01) were significantly associated with a decreased OS (Tables 1 and 3), which were used to generate the nomogram.

**Table 2:** Comparison of demographics of the derivation and validation sets

Item	Training cohort (*n* = 1090)	Validation cohort (*n* = 728)	*p* value
Age, years (mean ± SD)	61.56 ± 10.42	61.91 ± 9.44	0.48
Male [*n* (%)]	950 (87.2)	629 (86.4)	0.67
Race, White [*n* (%)]	1021 (93.7)	678 (93.4)	0.85
Histology [*n* (%)]			0.07
AC	827 (75.9)	526 (72.3)	
SRC	99 (9.1)	91 (12.5)	
ITAC	39 (3.6)	38 (5.2)	
MAC	46 (4.2)	27 (3.7)	
Other	79 (7.2)	46 (6.3)	
T category [*n* (%)]			0.74
T1	116 (10.6)	69 (9.5)	
T2	197 (18.1)	141 (19.4)	
T3	428 (39.3)	277 (38.0)	
T4	343 (31.4)	236 (32.4)	
No. of LNs dissected (mean ± SD)	16.27 ± 17.24	16.45 ± 16.41	0.83
Sites involved in the surgery [*n* (%)]			
Stomach	199 (18.3)	133 (18.3)	0.20
Stomach + esophagus	780 (71.5)	510 (70.1)	
Stomach + other organs ± esophagus	73 (6.7)	66 (9.1)	
PLN (mean ± SD)	3.30 ± 13.41	4.14 ± 15.49	0.22
LNR (mean ± SD)	0.13 ± 0.23	0.14 ± 0.24	0.50
LODDS (mean ± SD)	− 2.16 ± 1.42	− 2.14 ± 1.42	0.76

*AC* adenocarcinoma, *SRC* signet ring carcinoma, *ITAC* intestinal-type adenocarcinoma, *MAC* mucinous adenocarcinoma, *PLN* positive lymph node, *LNR* lymph node ratio, *LODDS* log odds of positive lymph nodes, *SD* standard deviation, *LNs* lymph nodes

**Table 3:** Cox regression coefficients and nomogram score

Cohort		Coefficients	Score
Age, years	≤ 65	0	0
	> 65	0.24	18.12
Grade	1/2	0	0
	3/4	0.27	20.56
PLN^a^		0.03	2.34 * PLN
LODDS^a^		0.15	11.11 * LODDS + 66.67

*PLN* positive lymph node, *LODDS* log odds of positive lymph nodes

^a^Continuous variables

### Construction, Comparison, and Validation of the Nomogram

**Fig. 2:**
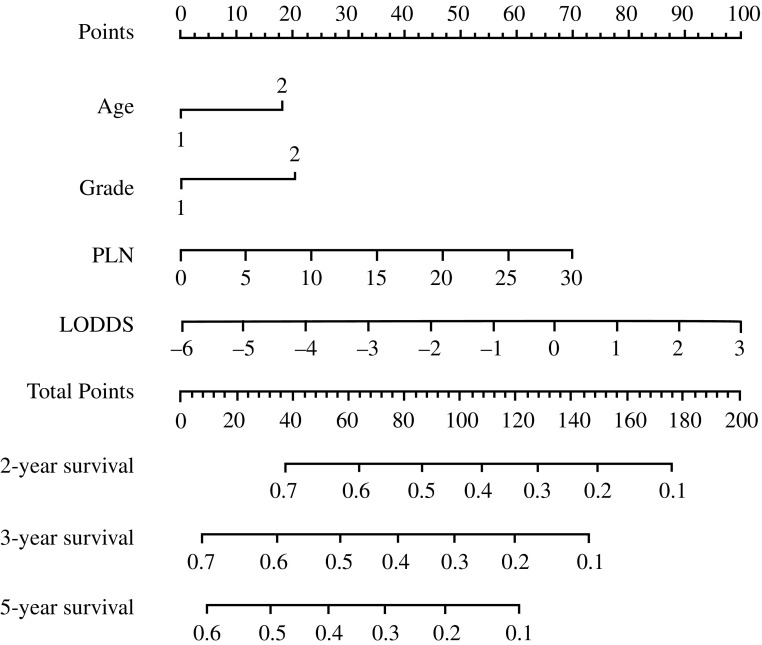
Nomogram construction in the training cohort. Nomogram for predicting the proportion of patients with 2-, 3-, and 5-year overall survival after the diagnosis of adenocarcinoma of the esophagogastric junction, based on four variables (age, grade, PLN and LODDS). The points for each variable are summed to give a total score from which the probability is estimated. *PLN* positive lymph node, *LODDS* log odds of positive lymph nodes

**Fig. 3:**
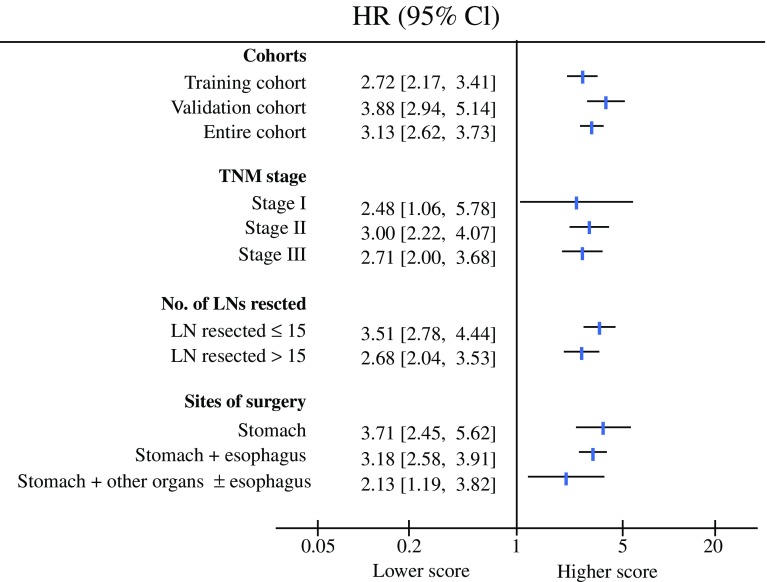
Survival impact of the nomogram in various subgroups. Forest plots of the associations between nomogram score and overall survival in various subgroups. Unadjusted HRs (boxes) and 95% CI (horizontal lines) are depicted. *HR* hazard ratio, *CI* confidence interval, *LNs* lymph nodes

**Fig. 4:**
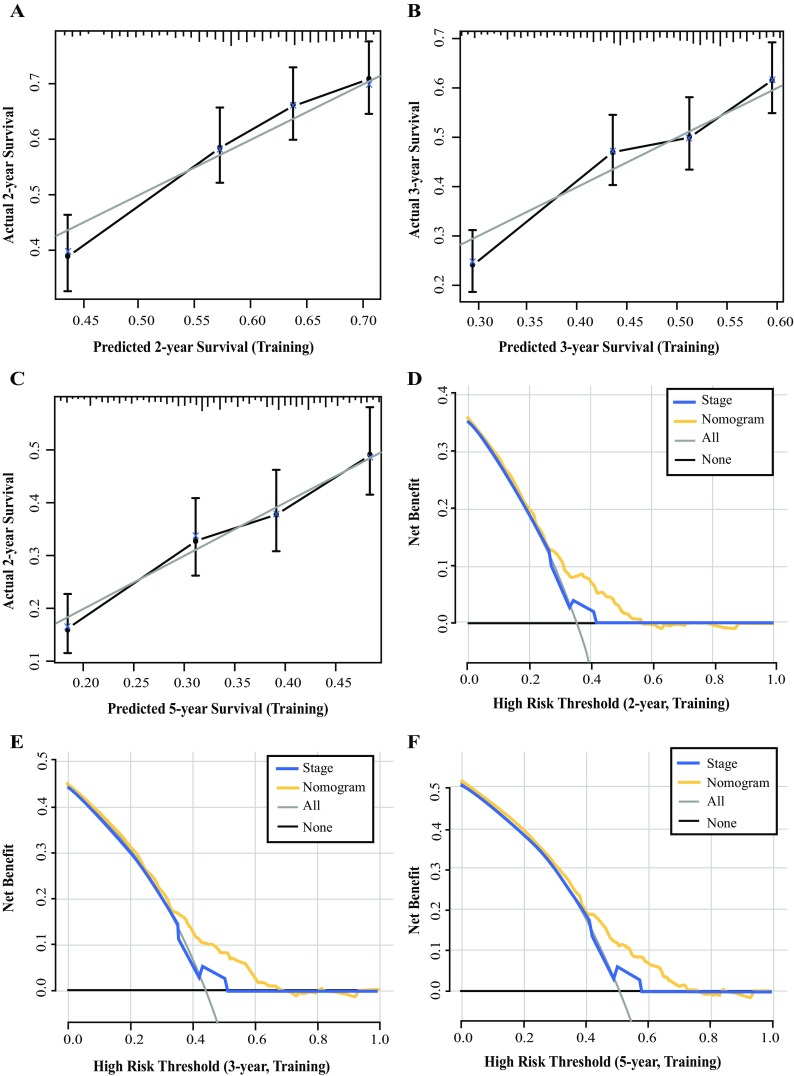
Calibration plots and decision curve analyses of the nomogram in the training cohort. **a**–**c** Plots depict the calibration of nomograms in terms of agreement between predicted and observed 2-, 3-, and 5-year outcomes in the training cohort. **d**–**f** Decision curve analyses of the nomogram and TNM stage for 2-, 3-, and 5-year risk in the training cohort

The nomogram can be used to provide a quantitative method to predict the probability of patient survival. As shown in Fig. 2, the nomogram indicated that the LODDS value was the largest contributor to prognosis, followed, in descending order, by PLN number, age, and Lauren classification. Each value or subtype within these variables was assigned a score on the points scale. By calculating the total score and locating this value on the total points scale, we were able to easily determine the estimated probability of 2-, 3-, and 5-year survival. The forest plots (Fig. 3) revealed that the nomogram could identify patients with different prognoses in either the training cohort, validation cohort, or entire cohort, in each TNM stage subgroup, regardless of the number of LNs resected or the site involved in the surgery. The calibration plots depicted in Fig. 4a–c and Fig. S1a–f showed that the derived nomogram performed well when compared with the performance of an ideal model using the training cohort, validation cohort, or entire cohort. Similarly, using DCA (Fig. 4d and e, and Fig. S2a–f), the nomogram also showed higher net benefit than TNM stage across a range of risk thresholds. We also compared the Harrell’s C-index of the nomogram with that of TNM staging. In the training cohort (Table 4), the C-index for the established nomogram for predicting OS in patients was significantly higher than that of the TNM staging system. Additionally, the AIC value of the nomogram also showed a higher prognostic value than TNM stage (Table 4), indicating that the nomogram, considering age, tumor grade, PLN, and LODDS, was the most effective tool in determining the prognosis of GCC patients who underwent preoperative radiotherapy. Similar results were also observed in the validation cohort as well as the entire cohort (Table 4).

**Table 4:** Comparison of the discrimination ability between the various LN classifications and staging models as determined by the Akaike Information Criterion test

Cohort		Stage 8th	Nomogram
Training	Log likelihood	3628.69	3210.29
	AIC	7261.38	6348.45
	C-index (95% CI)	0.55 (0.53–0.57)	0.61 (0.58–0.64)
Validation	Log likelihood	2145.23	1891.29
	AIC	4294.46	3736.24
	C-index (95% CI)	0.57 (0.55–0.59)	0.64 (0.61–0.67)
Entire	Log likelihood	6416.68	5686.47
	AIC	12,837.36	11,236.24
	C-index (95% CI)	0.56 (0.54–0.58)	0.62 (0.60–0.64)

*CI* confidence interval, *AIC* Akaike Information Criterion, *C*-*index* concordance index, *LN* lymph node

## Discussion

As patients with Siewert type II AEG have poor prognosis, investigation of novel therapeutic strategies, such as neoadjuvant chemotherapy with or without radiation, may be a reasonable approach to improve the management of these patients.^27^^–^31 The prognostic values of post-therapy clinicopathological factors in Siewert type II AEG patients undergoing preoperative radiotherapy have been investigated in several previous studies;^14^^,^^32^^–^35 however, these studies drew conflicting conclusions and all included both non-cardia (i.e. whole gastric) and cardia gastric cancer patients. To date, no studies have specifically focused on Siewert type II AEG patients. For example, in 2014, Orditura et al.^34^ analyzed 41 patients with AEG who underwent preoperative radiotherapy. With 54 months of follow-up, clinical response and postoperative TNM staging were demonstrated to be the only independent variables related to long-term survival. Ott et al.^35^ identified ypT category as an independent predictor in all AEG patients undergoing preoperative radiotherapy, as well as in patients classified as R0 responders, whereas ypN category did not maintain statistical significance in multivariate analysis. Furthermore, Gaca et al.^33^ reported that no variables were significantly associated with OS, and found that pathologic LN status was the only significant predictor of disease-free survival; however, the samples of both studies were small and lacked multivariate analyses.

To our knowledge, the present study is the first review of a large data set from a national cancer registry to explore the survival impact of postoperative clinicopathological factors in Siewert type II AEG patients undergoing preoperative radiotherapy. We found that ypT classification did not reach statistical significance in either univariate or multivariate analyses. Therefore, the AJCC staging system, which is based on depth of invasion and nodal status, might not be applicable for Siewert type II AEG patients after preoperative radiotherapy. In contrast, histology grade and postoperative LN status (PLN number and LODDS) were the only independent prognosis factors correlating with OS. Based on these findings, by integrating the beta coefficients of these independent variables, we established a nomogram for prognostic assessment in Siewert type II AEG patients after preoperative radiotherapy. We observed that the nomogram had a significantly better prognostic value than TNM stage alone.

LODDS is a newly reported index that is considered important and promising for prognosis assessment. The superiority of LODDS as a prognostic classification has been validated in various malignancies, including colorectal cancer, esophageal cancer, breast cancer, etc.^36^ In Siewert type II AEG, Xu^18^ performed a population-based study using the SEER database, suggesting that LODDS showed more accurate prognostic performance than PLN and LNR for post-surgery Siewert type II AEG, and could help in detecting survival heterogeneity for patients with no positive LNs involved. In agreement with these previous studies, we found that LODDS was also an independent factor for Siewert type II AEG patients who received preoperative radiotherapy. No doubt our findings further broaden the scope of the clinical use of LODDS.

Our study has some limitations. First, information regarding Siewert types I and III AEG were not available from the SEER database, which limited further analysis of other cancer subtypes. Second, it has been previously reported that clinical response to neoadjuvant therapy is another independent risk factor for survival after curative resection of AEG;^35^^,^^37^ however, the tumor status of patients diagnosed prior to undergoing preoperative radiotherapy was not recorded in the SEER database, thus we were unable to estimate the response rate in our study. Additionally, information regarding other significant risk factors, such as gastrectomy surgical margin status and chemotherapy conduction, was also missing. Despite these limitations, we are confident that our findings will help elucidate the prognostic value of the proposed nomogram in Siewert type II AEG patients who have received preoperative radiotherapy.

## Conclusions

The present study demonstrated that the novel nomogram is a better prognostic determinant than other available staging systems in Siewert type II AEG patients after preoperative radiotherapy. This model could enable clinicians to estimate the survival of Siewert type II AEG patients in a more precise fashion. Further prospective studies are required to validate our results.

## Electronic supplementary material

Below is the link to the electronic supplementary material.

Supplementary material 1 (PDF 208 kb)

Supplementary material 2 (PDF 184 kb)

Supplementary material 3 (DOCX 26 kb)
