# Supplementary figures and images for: Proposal of a Nomogram for Predicting Survival in Patients with Siewert Type II Adenocarcinoma of the Esophagogastric Junction After Preoperative Radiation
Source: Ann Surg Oncol. 2019 Feb 25;26(5):1292–300. doi: 10.1245/s10434-019-07237-7 (PMC6456486; doi:10.1245/s10434-019-07237-7)

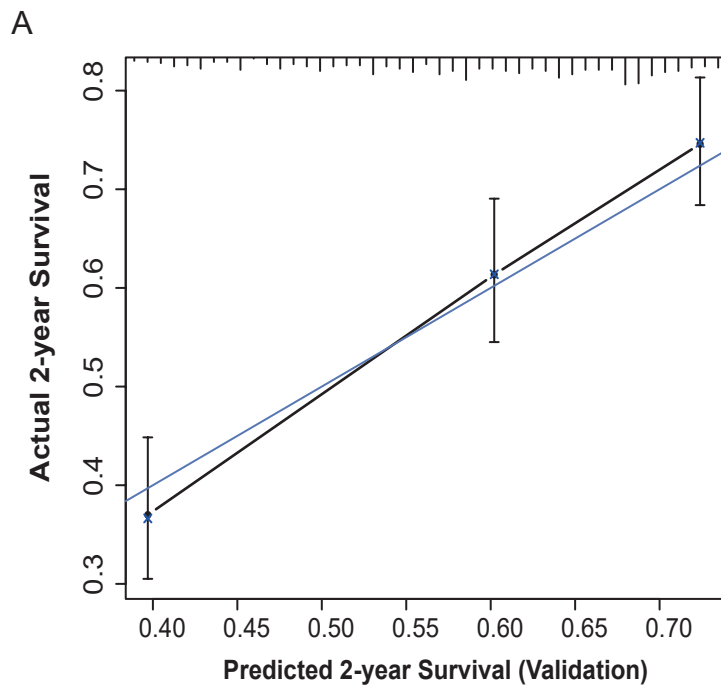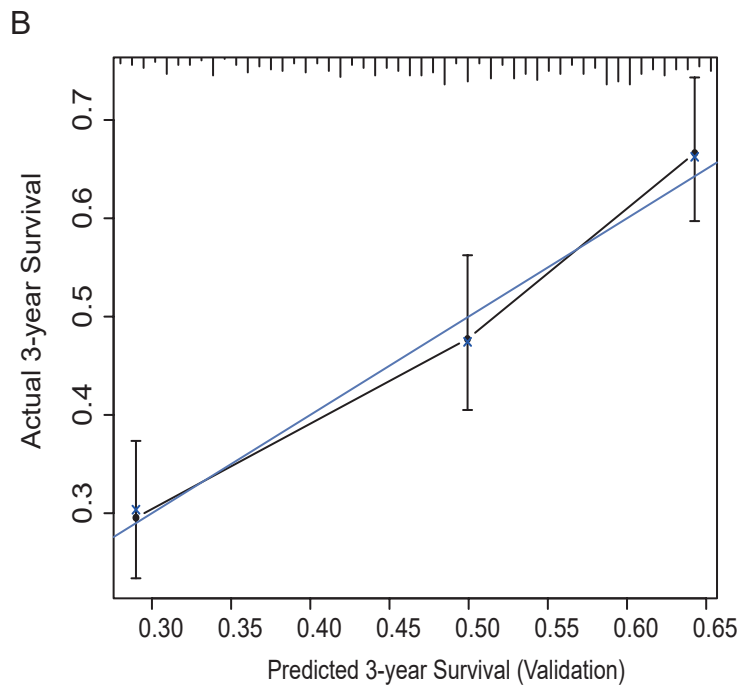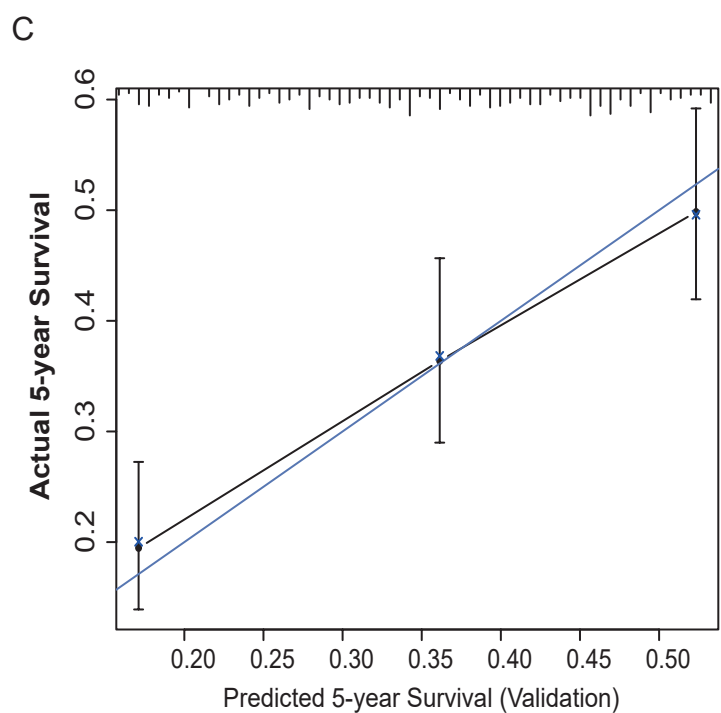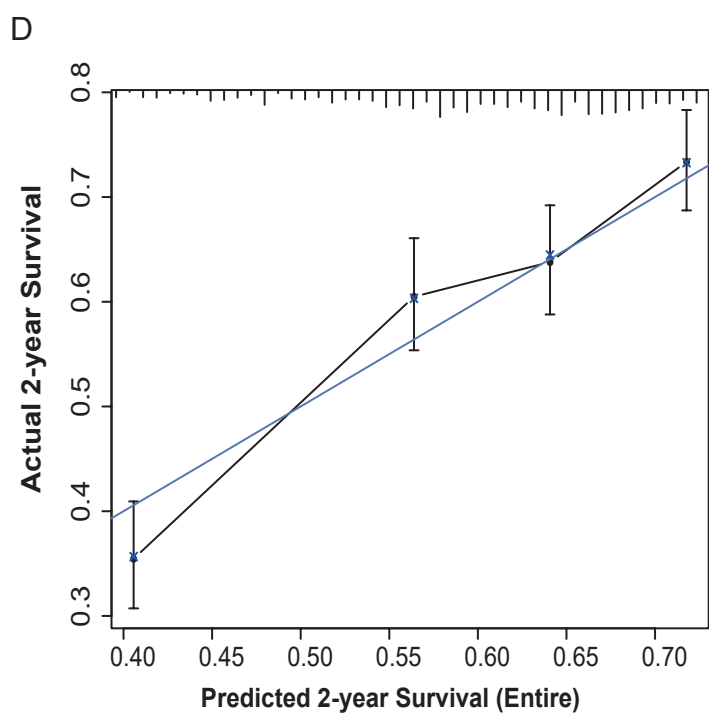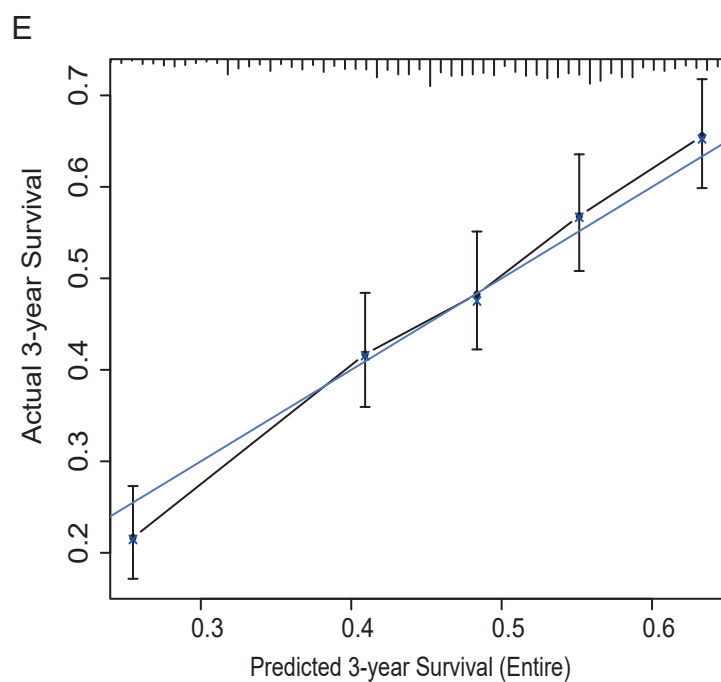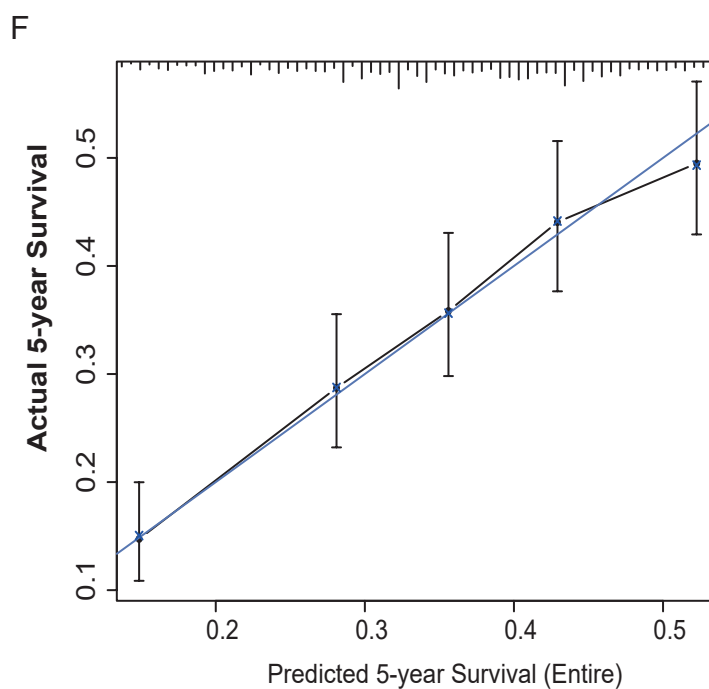

Supplement: Supplementary file 1 — Supplementary material 1 (PDF 208 kb) [file 10434_2019_7237_MOESM1_ESM.pdf]

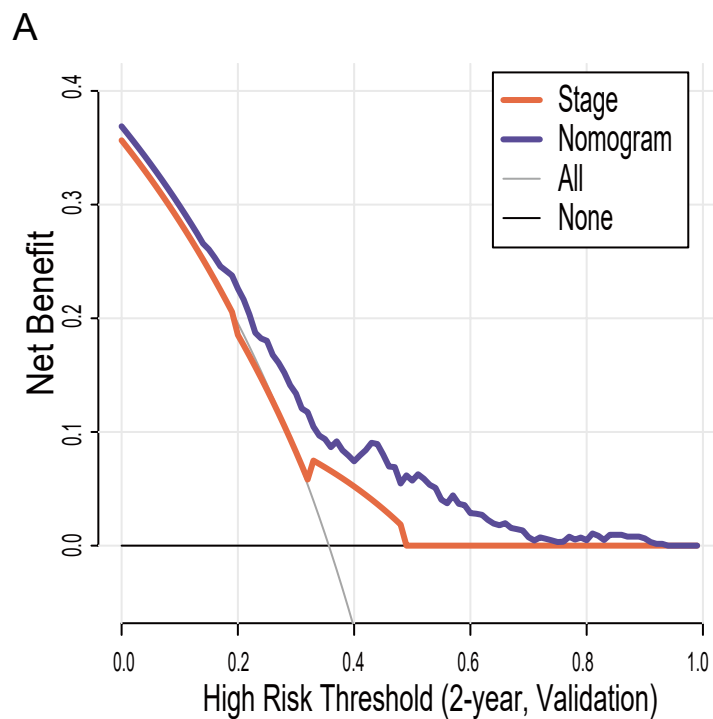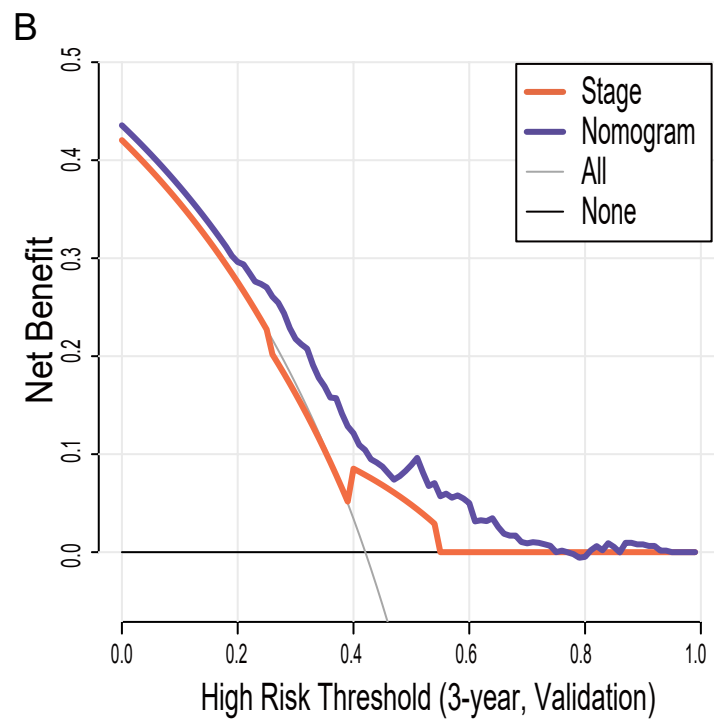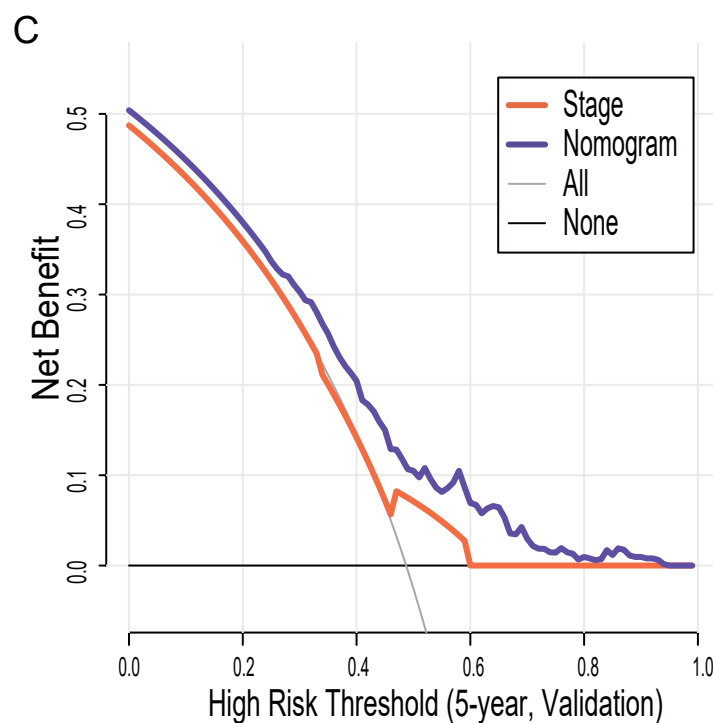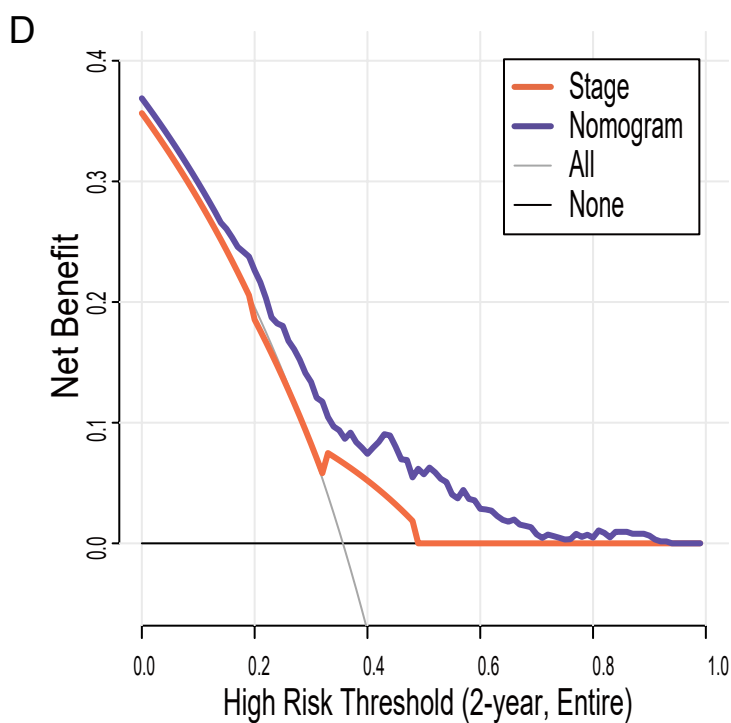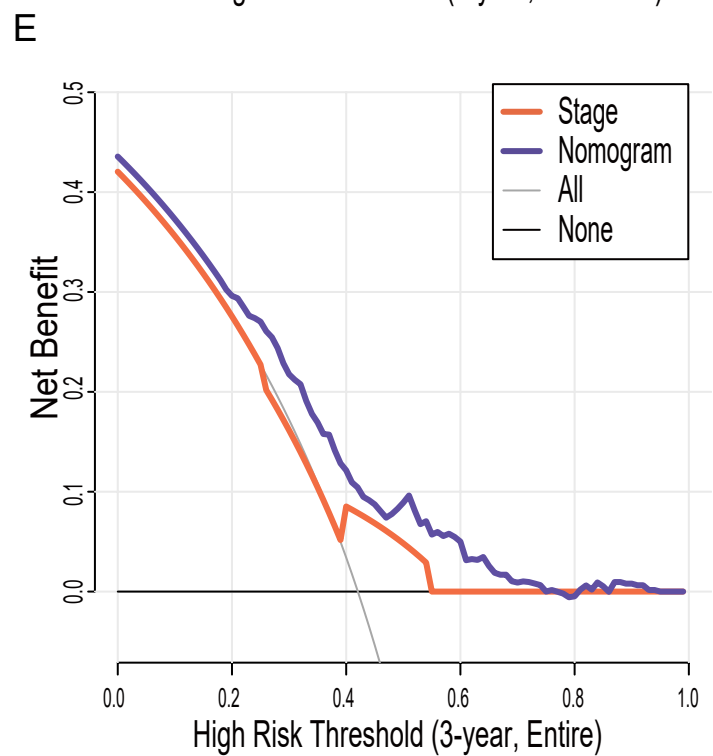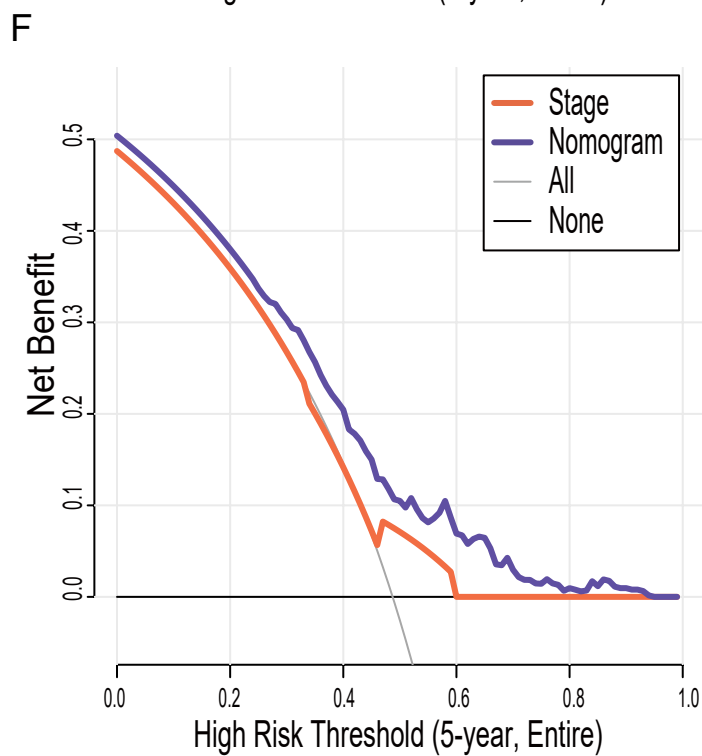

Supplement: Supplementary file 2 — Supplementary material 2 (PDF 184 kb) [file 10434_2019_7237_MOESM2_ESM.pdf]
