# Supplementary material for: Proposal of a Nomogram for Predicting Survival in Patients with Siewert Type II Adenocarcinoma of the Esophagogastric Junction After Preoperative Radiation
Source: Ann Surg Oncol. 2019 Feb 25;26(5):1292–300. doi: 10.1245/s10434-019-07237-7 (PMC6456486; doi:10.1245/s10434-019-07237-7)
Supplement: Supplementary file 3 — Supplementary material 3 (DOCX 26 kb) [file 10434_2019_7237_MOESM3_ESM.docx]

**Supplementary Results**

**Table S1.** Demographical and clinicopathologic variables definition

| **Variable** | **Bins created** |
| --- | --- |
| **Sex** | Male, Female |
| **Race** | White, non-White, |
| **Tumor grade** | Grade 1/2 (Well/Moderately differentiated), Grade 3/4 (Poorly/Undifferentiated) |
| **Histology type** | Unspecified AC, SRC, ITAC, MAC, Other |
| **ypT category** | ypT1, ypT2, ypT3, ypT4 |
| **PLN category** | N0 (0), N1 (1-2), N2 (3-6), N3 (> 7) |

Abbreviation: AC, adenocarcinoma; SRC, signet ring carcinoma; ITAC, intestinal type adenocarcinoma; MAC, Mucinous adenocarcinoma; PLN, positive lymph node

**Table S2.** Patients’ basic characteristics

|  |  | | **Number** | **Percent%** |
| --- | --- | --- | --- | --- |
| **Age** | ≤ 65 | | 1138 | 62.5 |
|  | 66+ | | 680 | 37.4 |
| **Sex** | Male | | 1579 | 86.9 |
|  | Female | | 239 | 13.1 |
| **Race** | White | | 1699 | 93.5 |
|  | Non-White | | 117 | 6.4 |
|  | Unknown | | 2 | 0.1 |
| **Grade** | Well/Moderately | | 694 | 38.2 |
|  | Poorly/Undifferentiation | | 935 | 51.4 |
|  | Unknown | | 189 | 10.4 |
| **Histology** | Unspecified AC | | 1353 | 74.4 |
|  | AC (Intestinal type) | | 77 | 4.2 |
|  | Signet ring carcinoma | | 190 | 10.5 |
|  | Mucinous AC | | 73 | 4.0 |
|  | Other types | | 125 | 6.9 |
| **ypT category** | ypT1 | | 185 | 10.2 |
|  | ypT2 | | 338 | 18.6 |
|  | ypT3 | | 705 | 38.8 |
|  | ypT4 | | 579 | 31.8 |
|  | Unknown | | 11 | 0.6 |
| **No. of LNs dissected** | 1-15 | | 1142 | 67.8 |
|  | >16 | | 676 | 37.2 |
| **ypN category** | ypN0 | | 1021 | 56.2 |
|  | ypN1 | | 407 | 22.4 |
|  | ypN2 | | 239 | 13.1 |
|  | ypN3 | | 111 | 6.1 |
|  | Unknown | | 40 | 2.2 |
| **Sites involved in the surgery** | Stomach | | 332 | 18.9 |
|  | Stomach + esophagus | | 1290 | 73.3 |
|  | Stomach + other orgams **±** esophagus | | 139 | 7.6 |
|  | Unspecified | | 57 | 3.1 |
| **Total** |  |  | 1818 | 100 |

Abbreviation: AC, adenocarcinoma; PORT, postoperative radiotherapy; PLN positive lymph node; LNR, lymph node ratio; LODDS, log **odds** of positive lymph nodes; NA, not available.
